# Endonasal-endoskopische anteriore Schädelbasischirurgie: Darstellung eines monozentrischen Entitätenprofils

**DOI:** 10.1007/s00106-024-01438-7

**Published:** 2024-02-23

**Authors:** Lisa Schmitz, Christian S. Betz, Katharina Stölzel

**Affiliations:** Klinik und Poliklinik für Hals‑, Nasen- und Ohrenheilkunde, Kopf- und Neurozentrum, Universitätsklinikum Hamburg-Eppendorf, Martinistr. 52, 20246 Hamburg, Deutschland

**Keywords:** Keimzell- und embryonale Tumoren, Otorhinolaryngologische chirurgische Verfahren, Chirurgische Endoskopie, Olfaktoriusneuroblastom, Nasennebenhöhlenchirurgie, Germ cell and embryonal neoplasms, Otorhinolaryngologic surgical procedures, Surgical endoscopy, Olfactory neuroblastoma, Paranasal sinuses

## Abstract

**Hintergrund:**

Die erweiterte endonasal-endoskopische Chirurgie („extended endoscopic endonasal surgery“, EEES) ist ein wesentlicher Bestandteil der Behandlung diverser pathologischer Veränderungen der vorderen Schädelbasis. Neben einer deutlichen Steigerung der Lebensqualität der betroffenen Patiente:innen und einem geringeren Komplikationsprofil als bei der offenen Schädelbasischirurgie sind die therapeutischen Ergebnisse bei richtiger Indikationsstellung vergleichbar.

**Material und Methoden:**

Es erfolgte eine retrospektive Datenerhebung aller endonasal-endoskopischen Schädelbasiseingriffe, die im Zeitraum von Juni 2018 bis November 2022 am universitären Schädelbasiszentrum Hamburg unter Führung der Klinik für Hals‑, Nasen- und Ohrenheilkunde durchgeführt wurden.

**Ergebnisse:**

Insgesamt 50 Fälle wurden identifiziert. Dabei handelte es sich in 56 % (28/50) um maligne Tumoren, in 24 % (12/50) um benigne pathologische Veränderungen mit direkter Schädelbasisbeteiligung sowie in 20 % (10/50) um anteriore Schädelbasisdefekte mit Rhinoliquorrhö. In 94 % (47/50) der Fälle konnte das präoperativ gesteckte Ziel des Eingriffs (repräsentative Biopsie, vollständige Resektion, Verschluss des Schädelbasisdefekts) erreicht werden. Komplikationen vom Grad III oder höher nach Clavien-Dindo traten in 4/50 Fällen auf. Im Beobachtungszeitraum wurden *n* = 5 Olfaktoriusneuroblastome diagnostiziert, von denen alle ausschließlich und erfolgreich endoskopisch operiert wurden.

**Schlussfolgerung:**

In den vergangenen Jahren hat sich das Spektrum der endoskopisch resezierbaren pathologischen Veränderungen der anterioren Schädelbasis stetig erweitert. Insbesondere mittellinienbezogene Tumoren wie das Olfaktoriusneuroblastom oder iatrogene/idiopathische Schädelbasisdefekte mit Liquorrhö werden mit sehr guten Ergebnissen vollständig endoskopisch therapiert. Nichtsdestotrotz ergeben sich auch Limitationen für diese Technik. Aufgrund der hohen Varianz des Umfangs frontobasaler Eingriffe, der Ausdehnung und der komplexen Anatomie sowie der sich überschneidenden Zuständigkeiten der Fachdisziplinen ist die Etablierung von zertifizierten Schädelbasiszentren und die Bündelung der frontobasalen Chirurgie an diesen Zentren von hoher Relevanz für die Qualitätssicherung.

**Video online:**

Die Online-Version dieses Beitrags (10.1007/s00106-024-01438-7) enthält ergänzendes Videomaterial.

## Abstract (en)

**Background:**

Extended endoscopic endonasal surgery (EEES) is an essential part of treatment of various pathologies of the anterior skull base. In addition to significant improvements in the quality of life of affected patients and a lower complication profile compared to open skull base surgery, the therapeutic results are comparable if the indications are correct.

**Materials and methods:**

Data of all endoscopic endonasal skull base procedures performed at the University Skull Base Center Hamburg under the direction of the Department of Otorhinolaryngology between June 2018 and November 2022 were retrospectively collected.

**Results:**

A total of 50 cases were identified. Of these, 56% (28/50) were malignant tumors, 24% (12/50) were benign pathologies with direct skull base involvement, and 20% (10/50) were anterior skull base defects with rhinoliquorrhea. In 96% (48/50) of cases, the preoperatively set goal of surgery (representative biopsy, complete resection, closure of the skull base defect) could be achieved. Complications grade III or higher according to Clavien–Dindo occurred in 4/50 cases. During the observation period, *n* = 5 olfactory neuroblastomas were diagnosed, all of which were exclusively and successfully operated on endoscopically.

**Conclusion:**

In recent years, the spectrum of endoscopically resectable pathologies of the anterior skull base has steadily expanded. In particular, midline-related tumors such as olfactory neuroblastoma or iatrogenic/idiopathic skull base defects with cerebrospinal fluid rhinorrhea are treated completely endoscopically with very good results. Nevertheless, there are also limitations to this technique. Due to high variance in the scope of frontobasal surgery, the extent, and the complex anatomy, as well as the overlapping responsibilities of the specialist disciplines, establishment of certified skull base centers and bundling of frontobasal surgery at these centers is highly relevant for quality assurance.

## Indikationsstellung

Die erweiterte endonasal-endoskopische Chirurgie („extended endoscopic endonasal surgery“, EEES) ist heute ein wesentlicher Bestandteil der multimodalen und interdisziplinären Therapie zahlreicher pathologischer Veränderungen der Nasenhaupt- und Nebenhöhlen mit Beteiligung der Schädelbasis oder intrazerebraler Ausdehnung [1, 13, 21–23]. Die Indikationsstellung wird hierbei ständig um neue Lokalisationen, auch in der mittleren und hinteren Schädelgrube, erweitert [17, 18, 29]. Das Komplikationsrisiko konnte dabei in den letzten Jahren durch das minimal-invasive Techniken deutlich gesenkt werden [13, 21]. Die etablierte Klassifikation nach Clavien-Dindo aus dem Jahr 2004 unterteilt operative Komplikationen dabei in 5 Grade, abhängig vom Interventionsbedarf und Einfluss auf das Patient:innenwohl [5].

Um insbesondere in diesem Feld seltener Erkrankungen und komplexer Anatomie Expertise zu bündeln und die Versorgungsqualität zu verbessern, etablieren sich zunehmend interdisziplinäre Schädelbasiszentren in der D‑A-CH-Region (Deutschland, Österreich und die Schweiz).

Ziel dieses Artikels ist es, das Entitätenprofil und die Relevanz eines nach den Kriterien der Gesellschaft für Schädelbasischirurgie e. V. zertifizierten Schädelbasiszentrums herauszuarbeiten und anhand konkreter Fallbeispiele näher zu beleuchten.

## Material und Methoden

Die Datenerhebung aller endonasal endoskopischer Schädelbasiseingriffe am Zentrum der Autoren erfolgte retrospektiv und ausschließlich qualitativ und deskriptiv. Eingeschlossen wurden Fälle von Juni 2018 bis November 2022, bei denen eine invasive, endoskopisch gestützte Biopsie erfolgte oder im Rahmen der hausinternen interdisziplinären Schädelbasiskonferenz eine endonasal endoskopische Resektion indiziert war. Darüber hinaus wurden Patient:innen mit endoskopisch Therapie einer Rhinoliquorrhö eingeschlossen.

## Ergebnis

### Entitätenprofil

Zwischen Juli 2018 und Oktober 2022 wurden 50 Fälle in die Fallserie aufgenommen (Tab. 1). Das Durchschnittsalter betrug 48,7 Jahre und variierte von 1 Tag bis 82 Jahre. Insgesamt waren 60 % der Patient:innen männlich und 40 % weiblich. Dabei wurden *n* = 28 bösartige Tumoren, *n* = 12 gutartige pathologische Veränderungen mit direkter Schädelbasisbeteiligung sowie *n* = 10 anteriore Schädelbasisdefekte mit Rhinoliquorrhö identifiziert.

**Table 1:** 

Entitätenprofil Schädelbasiszentrum UKE						
Klassifikation	Modalität	Entität	Anzahl	Prozedere	Gesetztes Therapieziel erreicht?	Komplikationen? (Major M/Minor m)
Benigne	Resektion/Entlastung	Meningeom	4	2/4 STR 2/4 PR + vorher TKR	Ja 4/4	Nein 4/4
		Meningo(enzephalo)zele	2	2/2 TR	Ja 2/2	Ja 1/2 (M)
		Hypophysenadenom	1	1/1 TR	Ja	Ja (M)
		Schwannom	1	1/1 STR + Caldwell-Luc	Ja	Nein
		Mukozele	1	1/1 TR	Ja	Nein
		Neurosarkoidose	1	1/1 PR	Ja	Nein
		Orbitaspitzenabszess	1	1/1 E	Ja	Nein
	Biopsie	Chondromesenchymales Hamartom	1	1/1 KE	Ja	Nein
Maligne	Resektion	Olfaktoriusneuroblastom	5	5/5 TR	Ja 5/5	Nein
		Sarkom	4	3/4 STR 1/4 PR	Ja 3/4 Nein 1/4 PR statt STR	Nein 4/4
		Chordom*	3	2/3 PR 1/3 STR	Ja 3/3	Nein 3/3
		Sinunasal undifferenziertes Karzinom	2	1/2 TR 1/2 STR statt TR	Ja 1/2	Ja 1/2 (M)
		Nasopharyngeales Adenokarzinom	1	1/1 TR	Ja	Nein
		Mukosales Melanom	1	1/1 PR (mehrfach)	Ja	Nein
	Biopsie	Metastase	3	Biopsie	Ja	Nein
		Lymphom	1		Ja	Nein
		NUT-Mittellinienkarzinom	1		Ja	Nein
		Myoepitheliales Karzinom	1		Ja	Nein
		Fortgeschrittene sinunasale Karzinome (PECA, SNUC, AC)	6		Ja	Nein
Rhinoliquorrhö	Deckung	Rhinoliquorrhö	10	9/10 dicht 1/10 VP-Shunt, dann dicht	Ja 9/10 Nein 1/10	Ja 1/10 (M)
*Gesamt*			*50*	*–*		

*AC *Adenokarzinom,* E* Entlastung, *KE *kombinierter Eingriff (offen und endoskopisch), *NUT* „nuclear protein in testis“, *PECA *Plattenepithelkarzinom,* PR* partielle Resektion, *SNUC *sinunasal undifferenziertes Karzinom,* STR* subtotale Resektion, *TKR* transkranielle Resektion, *TR* Totalresektion, *VP-Shunt* Ventrikuloperitonealshunt

*Chordome sind formell benigne Raumforderungen, werden in dem vorliegenden Fall aufgrund des osteodestruktiven Wachstums und des radikalen Therapieansatzes jedoch zu den malignen Erkrankungen gezählt

Insgesamt wurde in 94 % (47/50) der Fälle das initial geplante Operationsziel erreicht (Tab. 1). Nur in 6 % (3/50) der Fälle konnte das anvisierte Ziel nicht erreicht werden. In einem Fall erfolgte lediglich eine partielle Sarkomresektion mit postoperativem R2-Status. In einem weiteren Fall zeigte sich ein primär insuffizient mittels EEES verschlossener Schädelbasisdefekt. Hier gelang ein Verschluss erst nach operativer Revision sowie anschließender Anlage eines ventrikuloperitonealen Shunts. Zuletzt musste bei der endoskopischen Resektion eines sinunasal undifferenzierten Karzinoms (SNUC) aufgrund einer Komplikation vom Grad IV nach Clavien-Dindo [5] mit akzidenteller Eröffnung der A. carotis interna am vorderen Knie und konsekutiver starker Blutung die Resektion abgebrochen werden. Nach erfolgreichem endovaskulärem Stenting zeigte sich jedoch kein postoperatives neurologisches Defizit. Aufgrund des anschließend deutlich erhöhten perioperativen Risikos erfolgte keine erneute Nachresektion, sondern die Indikation zur adjuvanten Volldosis-Radiochemotherapie als individuellem Therapiekonzept. Es ereigneten sich in 3 weiteren Fällen Komplikationen vom Grad II mit weiterem medikamentösem Therapiebedarf: Sowohl nach Resektion einer Meningoenzephalozele als auch nach Deckung eines Schädelbasisdefekts entwickelte sich eine postoperative Meningitis, die jeweils erfolgreich antibiotisch und ohne bleibende Schäden therapiert werden konnte. In einem weiteren Fall trat nach der Resektion eines großen, die Nasenhaupthöhle vollständig verlegenden Hypophysenadenoms postoperativ eine unilaterale Abduzenzparese auf, bei der sich im Verlauf einiger Wochen unter oraler Steroidtherapie eine Komplettremission zeigte.

Auch wenn es sich bei der folgenden Darstellung um ein ungewöhnliches Format handelt, sind die Autoren davon überzeugt, dass es diese Form ermöglicht, einen guten Gesamtüberblick über das Entitätenprofil eines Schädelbasiszentrums zu erlangen und gleichermaßen auch die konkreten Abläufe zu beleuchten. Aus diesem Grund wird im Folgenden die Gruppe der Olfaktoriusneuroblastome (ONB) exemplarisch dargestellt und anhand eines Fallbeispiels das Prozedere genauer erläutert. Auf eine weitere differenzierte Beschreibung der anderen Entitäten wird an dieser Stelle aus Platzgründen bewusst verzichtet.

### Olfaktoriusneuroblastome

Von den 5 Patient:innen mit diagnostiziertem ONB (Tab. 2) waren 3 männlich und 2 weiblich. Das Alter bei der Erstdiagnose betrug im Mittel 50,4 Jahre (Spannbreite 32–64 Jahre). Alle Tumoren wiesen mindestens eine Arrosion der Lamina cribrosa (T2) auf, und bei keinem der Patient:innen konnten regionäre oder Fernmetastasen diagnostiziert werden. Die Resektion umfasste die Entfernung von mindestens einem Bulbus, in 2 Fällen beider Bulbi olfactorii (T3). Die komplikationslose Rekonstruktion erfolgte in 4 von 5 Fällen durch einen nasoseptalen Lappen (NSF). Im Rahmen des interdisziplinären Schädelbasisboards wurde für 4 von 5 Patiente:innen eine adjuvante Strahlentherapie (RT) indiziert. In 2 Fällen erfolgte eine Schwerionenbestrahlung (C12) und in 2 Fällen eine perkutanen Strahlentherapie („external beam radiation“, EBR) mit Gamma-Strahlung. Alle Patient:innen wiesen bis zum Zeitpunkt der Veröffentlichung keine Anzeichen eines Rezidivs auf, wobei das mittlere Follow-up bei 25,4 Monaten lag. Zur Veranschaulichung des konkreten Prozedere am universitären Schädelbasiszentrum des Universitätsklinikums Hamburg-Eppendorf wird ein Fall im Folgenden detailliert dargestellt.

**Table 2:** 

Olfaktoriusneuroblastome						
Staging	Grading	Resektion	Rekonstruktion	Komplikationen	Adjuvanz	Rezidivfreies Überleben in Monaten
(UISS)	(Hyams)					
pT4 cN0 cM0	II	TR inkl. intrakranieller Anteile und bilateraler Bulbi olfactorii	NSF	Keine	RT (C12)	19
cT2 cN0 M0	II	TR	NSF	Keine	Keine	19
pT2 cN0 cM0	II	TR inkl. Lamina cribrosa	Kollagenvlies	Keine	RT (EBR)	28
pT3 cN0 cM0	I	TR inkl. unilateralem Bulbus olfactorius	NSF	Keine	RT (EBR)	25
pT3 cN0 cM0	II	TR inkl. intrakranieller Anteile und bilateraler Bulbi olfactorii	NSF	Keine	RT (C12)	36

*C12* Schwerionentherapie, *EBR* „external beam radiation“, *NSF* „nasoseptal flap“, *RT* Radiotherapie, *TR* Totalresektion, *UISS* UCLA Integrated Staging System

### Fallbeispiel: Olfaktoriusneuroblastom im Stadium cT4 cN0 cM0

**Figure 1:**
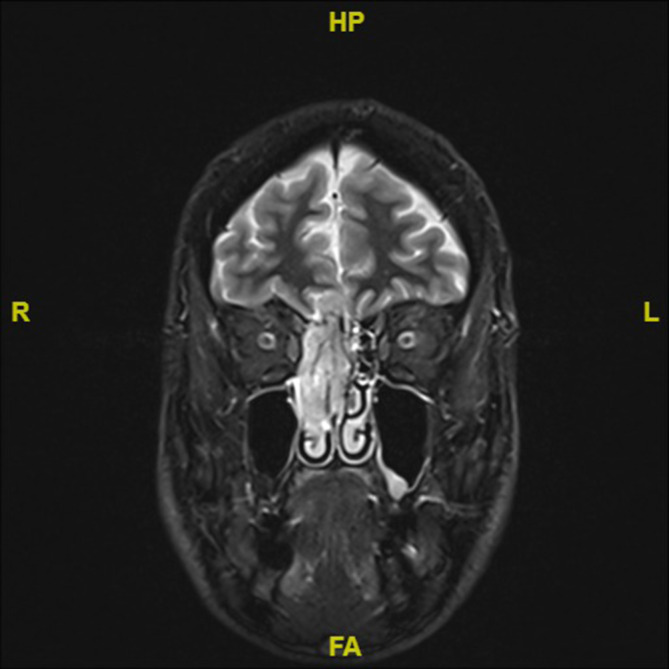

Initial erfolgte die Vorstellung aufgrund einer progredienten rechtsseitigen Nasenatmungsbehinderung sowie einer rechtsseitigen, klaren Rhinorrhö. Eine Computertomographie (CT) zeigte einen raumfordernden Prozess der rechten Nasenhaupthöhle. In der anschließenden Magnetresonanztomographie (MRT) des Kopfs (Abb. 1) erhärtete sich aufgrund der Beteiligung von Schädelbasis und intrazerebralen Strukturen der Verdacht einer malignen Raumforderung, sodass daraufhin die Vorstellung in der Klinik der Autoren stattfand.

Nach histologischer Diagnosesicherung eines ONB erfolgte ergänzend die Vervollständigung der Staging-Untersuchung mittels CT von Thorax und Abdomen. Insgesamt ließ sich ein Tumor im Stadium cT4 cN0 cM0 und vom Grad II nach Hyams nachweisen. Im Rahmen der interdisziplinären Schädelbasiskonferenz wurde im Anschluss die Indikation zur Resektion mittels EEES gestellt. Eine optionale Erweiterung durch einen offenen Zugang bei unzureichender endoskopischer Adressierbarkeit wurde präoperativ ebenfalls aufgeklärt. Außerdem erfolgte die präoperative dreidimensionale Planung mittels vorhandener fusionierter Bildgebung des Kopfs. Letztlich gelang eine ausschließlich endonasal-endoskopische Resektion des ONB (Video) durch die Anwendung eines optischen Navigationssystems.

Intraoperativ wurde zunächst nach Entfernung der Tumormassen der Nasenhaupthöhle mittels Shaver bei nicht infiltriertem Septum ein linksseitiger nasoseptaler Lappen zur späteren Defektdeckung gehoben. Die Lamina papyracea rechts zeigte sich teilweise infiltriert, wohingegen die Periorbita sich in den Schnellschnitten unauffällig zeigte. Für die Resektion der infiltrierten Lamina cribrosa inklusive beider Bulbi olfactorii und der duralen sowie intrazerebralen Anteile erfolgte die interdisziplinäre Zusammenarbeit mit der Neurochirurgie. Entnommene Schnellschnitte der endgültigen Absetzungsränder stellten sich negativ dar. Daraufhin konnte die gemeinsame mehrschichtige Defektdeckung durchgeführt werden. Der intrazerebrale Defekt wurde zunächst mittels Muskelplombe aus dem M. vastus lateralis aufgefüllt und der Defekt anschließend mittels vorher gehobenem nasoseptalen Lappen („Hadad-Bassagasteguy flap“) gedeckt.

Intra- und postoperativ zeigte sich kein Anhalt für eine Liquorleckage, und negative Resektionsränder wurden histologisch bestätigt. Es schloss sich eine adjuvante Schwerionentherapie der ehemaligen Tumorregion inklusive Sicherheitssaum im Schwerionenzentrum Heidelberg an. Nach 12 Monaten Follow-up zeigt sich bisher weiterhin kein Anhalt für ein lokoregionäres Rezidiv in der regelmäßigen Nachsorge.

## Diskussion

Die vorgestellte Fallserie zeigt die große Bandbreite der Anwendung der EEES an einem zertifizierten universitären Schädelbasiszentrum in Deutschland. Anhand des Fallbeispiels können sowohl die wesentlichen Vorteile dieser Operationstechnik als auch die Relevanz der Etablierung eines multidisziplinären Schädelbasiszentrums veranschaulicht werden. Darüber hinaus beleuchtet es auch die aktuellen Grenzen und Hürden, mit denen dieses junge Spezialgebiet aktuell konfrontiert ist.

### Präoperative Planung

Der gewählte Fall veranschaulicht die Relevanz einer exakten, auf den Bildgebungen basierenden präoperativen Planung für den Verlauf einer Operation. Mithilfe von dreidimensionalen Bildern kann zuvor die exakte Lagebeziehung zu relevanten Strukturen wie etwa der A. carotis interna innerhalb der Schädelbasis sowie einer möglichen intrazerebralen Ausdehnung eruiert werden. Anhand dessen kann dann bereits vor Beginn der Operation eine interdisziplinäre Zusammenarbeit mit der Neurochirurgie gebahnt werden. In diesem Kontext können dann auch die Möglichkeiten der Defektdeckung wie beispielsweise ein nasoseptaler Lappen und mögliche „Rescue-Konzepte“ gemeinsam besprochen und geplant werden. Darüber können fusionierte Bilder eine „augmented reality neuronavigation“ auch während der Operation zu besserer Orientierung führen, wodurch das Resektionsergebnis verbessert und das Komplikationsrisiko minimiert werden kann [26].

### Vorteile der Endoskopie aus Chirurgensicht

Aufgrund der medianen Lage und des kegelförmigen Wachstums ist das ONB auch in fortgeschrittenen Stadien zumeist sehr gut für die EEES geeignet. Aufgrund der Seltenheit der Entität beruhen die Daten hauptsächlich auf Fallserien und retrospektiven Erhebungen. Es gibt jedoch Hinweise darauf, dass die endoskopische Therapie der offenen Operation hinsichtlich des Gesamtüberlebens und des rezidivfreien Überlebens überlegen ist [14, 27, 34]. Dies könnte darauf zurückzuführen sein, dass der endoskopische Ansatz dem offenen Ansatz bei der Sicherung negativer Resektionsränder überlegen zu sein scheint [14]. Dazu trägt auch die optimierte Übersicht über das Operationsgebiet bei einem endoskopischen Ansatz bei. Dafür sorgen neben Weitwinkel- und Winkeloptiken in Kombination mit hochauflösenden (4K-)Endokamerasystemen auch Elemente von z. B. 3‑D-Techniken [30], robotergestützten Verfahren [17] und „augmented reality“ [37]. Eine optimierte Übersicht ermöglicht eine präzise Visualisierung der Tumoransatzfläche sowie relevanter umliegender Strukturen. Dies ist bei einem offenen Zugang in diesem Umfang kaum zu erreichen [3]. Die klare Visualisierung der Schnittränder ist jedoch von übergeordneter Relevanz, da die R0-Resektion als einer der wichtigsten prognostischen Marker für das Langzeitergebnis gilt [6, 24, 33]. Die neueste Datenlage zeigt dabei zusätzlich, dass die Piecemeal-Technik der En-bloc-Resektion nicht unterlegen ist [3, 25].

### Nachteile

Jedoch ist bei der Durchführung endoskopischer Techniken zu beachten, dass eine teure, empfindliche und schnell veraltende technische Ausstattung sowie ein geringfügig höherer Personalaufwand als bei offenen Techniken zu gewährleisten ist. Zum anderen ist das Erlernen der notwendigen Fähigkeiten deutlich komplexer als beispielsweise bei der alleinigen Nebenhöhlenchirurgie und weist aus diesem Grund eine insgesamt flachere Lernkurve auf. Die intraoperative Darstellung umliegender, kritischer nervaler und vaskulärer Strukturen in einem endoskopischen Ansatz erfordert viel Erfahrung. Darüber hinaus gibt es auch Tumorlokalisationen, die endoskopisch nicht adäquat adressierbar sind. Dazu gehören insbesondere seitliche Abschnitte der Schädelbasis, eine orbitale Invasion, eine Beteiligung der Stirnhöhlen oder der Kieferhöhlenvorderwand, Hautbeteiligung und Arrosion oder Invasion größerer neurovaskulärer Strukturen. Ein weiterer großer Nachteil dieser Art der Resektion ist wohl v. a. die schwierigere histologische Beurteilung und Orientierung aufgrund der starken Fragmentierung [28]. Das vorliegende Fallbeispiel veranschaulicht, dass sowohl in der Diagnosesicherung als auch in der Schnellschnittdiagnostik eine engmaschige Kooperation mit der Pathologie erforderlich ist, da ansonsten eine Fehldeutung der Resektionsränder droht. Zum einen erfordert allein die Diagnosestellung seltener histologischer Entitäten viel Erfahrung. Durch die Bündelung von Expertise an zertifizierten Zentren und in spezialisierten Netzwerken kann die Diagnosestellung, beispielsweise auch mittels zügiger Referenzpathologien, verbessert werden.

Zu Beginn der Ära der endoskopischen Chirurgie der Rhinobasis traten darüber hinaus noch in bis zu 40 % der Fälle postoperative Liquorleckagen auf, die nicht selten mit weiteren schweren Komplikationen verbunden waren [12]. In jüngster Zeit wurde in diesem Bereich eine deutliche Reduktion erreicht. Dies ist größtenteils auf die Verwendung autologer lokaler Lappenplastiken, wie dem nasoseptalen Lappen und anderer verfügbarer Materialien wie Kollagen-Vlies-Produkte, zurückzuführen. Hierdurch konnte die postoperative Liquorleckage auf etwa 5–10 % reduziert werden [2, 16]. Es wurde außerdem gezeigt, dass eine kombinierte Underlay- und Onlay-Technik die besten Ergebnisse hinsichtlich der Verschlussrate und des rezidivfreien Überlebens aufweist. Auch in dem vorliegenden Fall hat die Kombination dieser Verfahren zu einem komplikationslosen Verlauf beigetragen.

### Vorteile aus Patient:innensicht

Besonders hervorzuheben ist darüber hinaus die verbesserte Lebensqualität der Patient:innen, da das minimal-invasive Vorgehen aufgrund eines kleineren Wundgebiets die Schmerzintensität senken und die Heilung fördern kann. Neben einem kosmetisch besseren Ergebnis und dem besseren Erhalt funktionell relevanter Strukturen [1, 11] ist die EEES auch mit einer geringeren Komplikationsrate wie beispielswiese postoperativen Blutungen assoziiert [13, 21]. Dies korreliert mit den Ergebnissen der Datenerhebung durch die Autoren. In Studien konnte bewiesen werden, dass diese Vorteile sich auch in einer halbierten Hospitalisierungszeit von nur noch 3–6 Tagen widerspiegeln [8, 11, 13, 19].

So kann insbesondere der Zeitraum zwischen Resektion und adjuvanter Therapie durch den Einsatz von EEES deutlich verkürzt werden. So zeigten Xiao et al. eine Verkürzung dieses Zeitraums von 3 Monaten auf 15 Tage [36]. In der Literatur gilt eine adjuvante Radiatio dabei als dringlich erforderlich. Dies ist v. a. der Tatsache geschuldet, dass häufig nur ein geringer Sicherheitsabstand zur Schonung wichtiger anatomischer Strukturen (z. B. des Orbitainhalts) gewählt wurde. Aufgrund dessen und eines insgesamt unübersichtlichen Tumorgebiets ist eine tatsächliche mikroskopische R0-Resektion häufig nicht definitiv zu bestätigen. Innerhalb der adjuvanten Radiatio ist die Datenlage zu verschiedenen Modalitäten jedoch unzureichend, sodass hier bisher keine tragfähige Empfehlung ausgesprochen wurde. Erste Ergebnisse deuten auf eine gute Wirksamkeit mit 2‑Jahres-Überlebensraten von 80–90 % bei der Schwerionentherapie auch für fortgeschrittene ONB hin [15, 31].

In den vorliegenden Fällen ergibt sich bisher kein Hinweis auf ein Rezidiv. Dies könnte damit zusammenhängen, dass die Prognose von niedriggradigen (Hyams-Grade I und II) ONB, wie in den hier vorliegenden Fällen, mit 56–80 % deutlich höher ist als die von hochgradigen ONB, die bei 20–40 % liegt [35].

### Relevanz spezialisierter Schädelbasiszentren

Alles in allem bestätigt die aktuelle Literatur, dass die EEES in vielen Fällen eine gleichwertige Alternative zu einem offenen Zugang ist und vielversprechende Ergebnisse in Bezug auf das Gesamtüberleben und die Reduktion der Lokalrezidivrate zeigt [7]. Doch trotz der stetigen Erweiterung des Indikationsprofils endoskopischer Zugänge und ihrer klaren Vorteile bleiben weiterhin viele Indikationen für eine offene Technik bestehen [22]. Für die Erzielung gleichwertiger onkologischer Ergebnisse bedarf es somit einer sorgfältigen Patient:innenselektion [17, 21], da das wichtigste prognostische Kriterium bei Malignomen eine realisierbare R0-Resektionssituation ist [4].

Jedoch unterstreicht internationale und multidisziplinäre Konsenserklärungen, dass die endoskopische Schädelbasischirurgie nach einer umfangreichen Literaturauswertung nur einen bescheidenen Evidenzgrad mit großen Lücken aufweist. Vor allem prospektive Studien und systematische Übersichten sind derzeit noch selten. Dies ist jedoch eine Grundvoraussetzung, um in Zukunft die Therapie zunehmend auf evidenzbasierte Entscheidungen zu stützen [34].

Da pathologische Veränderungen der Schädelbasis eine insgesamt seltene Gruppe in einer kleinen Patient:innenpopulation darstellen, sind sie oft kein Teil der täglichen klinischen Praxis. Dies erschwert sowohl die Diagnosestellung als auch den weiteren therapeutischen Weg. Insbesondere in kleineren Krankenhäusern wird eine optimale Versorgung beispielsweise durch mangelnde Erfahrung und das Fehlen von Spezialdisziplinen wie einer neurochirurgischen Abteilung oder einer interventionellen Neuroradiologie erschwert. Es wurde nachgewiesen, dass die regelmäßige Durchführung der EEES an Zentren der Maximalversorgung und die dadurch gewonnene Expertise und Erfahrung zu höheren R0-Resektionsraten [9, 20, 33] und einem verbesserten Gesamtüberleben [32] sowie zu geringeren Liquorleckageraten nach Rekonstruktion führen [10]. Aus diesem Grund halten die Autoren die Einrichtung von spezialisierten Schädelbasiszentren für eine bestmögliche Versorgung für unerlässlich. Hierbei sollte eine Zertifizierung als Schädelbasiszentrum durch die Gesellschaft für Schädelbasischirurgie e. V. angestrebt werden.

## Fazit für die Praxis

- Anhand der hier dargestellten Daten und der verfügbaren Literatur stellt sich die erweiterte endonasal-endoskopische Nasennebenhöhlenchirurgie (EEES) für eine Vielzahl von pathologischer Veränderungen der vorderen Schädelbasis als geeignete chirurgische Technik dar.
- Aufgrund der Seltenheit der Entitäten, der hohen Komplexität von Anatomie und Operation sowie der erforderlichen multidisziplinären Patient:innenversorgung sollte sie in spezialisierten Zentren stattfinden.
- Eine Zertifizierung nach den Kriterien der Gesellschaft für Schädelbasischirurgie oder vergleichbaren Qualitätsstandards ist anzustreben.
- Eine kontinuierliche Überprüfung der Erfolgs- und Komplikationsraten sollte integraler Bestandteil sein.

### Supplementary Information





## Abkürzungen

AC: Adenokarzinom
ACT: Adjuvante Chemotherapie
ART: Adjuvante Radiotherapie
C12: Schwerionentherapie
CT: Computertomographie
EBR: „External beam radiation“
EES: Endonasal-endoskopische Schädelbasischirurgie
KE: Kombinierter Eingriff
MRT: Magnetresonanztomographie
NCT: Neoadjuvante Chemotherapie
NSF: Nasoseptaler Lappen („flap“)
OE: Offener Eingriff
ONB: Olfaktoriusneuroblastom
PECA: Plattenepithelkarzinom
PR: Partielle Resektion
PST: Palliative Systemtherapie
RCT: Radiochemotherapie
RT: Radiotherapie
SNUC: Sinunasal undifferenziertes Karzinom
STR: Subtotale Resektion
TKR: Transkranielle Resektion
TR: Totale Resektion
T‑VEC: Talimogen-Laherparepvec
UISS: UCLA Integrated Staging System
